# Значимость и методология мониторинга кальциемии при нарушениях минерального обмена: вызовы и перспективы

**DOI:** 10.14341/probl13413

**Published:** 2024-02-06

**Authors:** К. А. Чубакова, Е. М. Каменских, Т. В. Саприна

**Affiliations:** Сибирский государственный медицинский университет; Сибирский государственный медицинский университет; Сибирский государственный медицинский университет

**Keywords:** кальций, гипокальциемия, первичный гиперпаратиреоз, нарушения кальциевого обмена, устройства Point-of-Care, нательные электронные устройства

## Abstract

Нарушения кальций-фосфорного обмена могут вызывать тяжелые соматические патологии, требующие медикаментозной коррекции, в том числе в стационарных условиях. При этом распространенность заболеваний, сопровождающихся нарушениями кальциевого обмена, варьирует от низкой до средней. Например, первичный гиперпаратиреоз как одна из наиболее частых причин патологических изменений метаболизма кальция вследствие гиперсекреции паратиреоидного гормона встречается с частотой от 85 до 233 случаев на 100 тыс. человек. В странах, где рутинно измерение кальция крови не проводится, данное заболевание и сходные состояния диагностируются реже, и на более поздних стадиях с превалированием манифестных и осложненных форм. Однако нарушения кальциевого обмена требуют своевременного выявления и коррекции с целью предотвращения осложнений. При этом в ряде клинических ситуаций стандартный лабораторный анализ не является оптимальным диагностическим вариантом в связи с длительностью и сложностью проведения. В частности, развитие острой гипер- и гипокальциемии требует более быстрого получения результата исследования крови. Также при ведении больных с хроническими нарушениями метаболизма кальция, особенно на этапе подбора лекарственной терапии и титрации доз, перспективно применение способов определения уровня кальция, позволяющих оценить актуальный уровень кальциемии непосредственно на врачебном приеме. В связи с этим при необходимости длительного мониторинга или в экстренных ситуациях потенциальную пользу можно получить при использовании портативных устройств формата Point-of-Care (POC) или нательных биосенсоров (“wearable biosensors”). В данном обзоре рассмотрены клинические и методологические аспекты мониторинга уровня кальция, их возможности и практические ограничения, а также освещены перспективы разработки и внедрения устройств типа POC и биосенсоров, чувствительных к ионизированному кальцию.

## Abstract (en)

Disorders of calcium and phosphorus metabolism can cause severe complications that require changing of therapeutic strategies and a long treatment in a hospital. The prevalence of diseases accompanied by calcium metabolism disorders varies from low to moderate. For example, primary hyperparathyroidism, as one of the most common causes of pathological changes in calcium metabolism due to parathyroid hormone hypersecretion, occurs with a frequency of 85 to 233 cases per 100 thousand people. In countries where blood calcium measurements are not routinely carried out, this disease and similar conditions are diagnosed less frequently, and at later stages, with a predominance of manifest and complicated forms. However, calcium metabolism disorders require timely detection and correction in order to prevent complications. At the same time, in a number of clinical situations, standard laboratory analysis is not the optimal diagnostic option due to the duration and complexity of its implementation. In particular, the development of acute hyper- and hypocalcemia requires faster obtaining of blood test results. It is promising to apply technologies allowing to quick assess the current level of calcium directly at a doctor’s appointment especially in cases of drug doses adjustment for patients with chronic disorders of calcium metabolism. In this regard, when long-term monitoring of calcemia is required or in emergency situations, the potential benefit can be obtained by using portable Point-of-Care (POC) devices or wearable biosensors. This review examines the clinical and methodological aspects of monitoring calcium levels, their capabilities and practical limitations, and also highlights the prospects for the development and implementation of POC devices and biosensors for ionized calcium.

## ВВЕДЕНИЕ

Уровень внеклеточного кальция в биологических жидкостях человека является константой, поддерживаемой в довольно узком диапазоне, и регулируется несколькими системами организма [1]. Гомеостаз кальция тесно связан с секрецией паратиреоидного гормона (ПТГ), метаболизмом фосфора и эффектами витамина D [2]. Ионы кальция участвуют в механизмах передачи нервного импульса, мышечных сокращениях, свертывании крови, межклеточной адгезии и секреции ряда гормонов [3]. В связи с этим изменение баланса кальция, фосфора, ПТГ и витамина D обуславливает гипер- или гипокальциемию и приводит к мультисистемным нарушениям [4]. При резких колебаниях концентрации кальция крови возможно развитие острых состояний: гипо- и гиперкальциемических кризов [5][6]. Менее выраженное отклонение уровня кальция от референсных значений при длительном течении патологии активирует компенсаторные механизмы, в связи с чем клиническая картина в таких случаях может быть стертой, вплоть до бессимптомной [5]. Однако осложнения хронического нарушения фосфорно-кальциевого обмена могут стать причиной стойких нарушений функций организма с ограничением трудоспособности, в частности — низкотравматичных переломов, почечной недостаточности (стадии 4–5 хронической болезни почек (ХБП), особенно в сочетании с нефролитиазом) [3]. В связи с этим важно своевременное определение уровня кальция, что позволит подобрать оптимальную стратегию лечения. Для оценки кальциемии в неотложных ситуациях или при необходимости ее длительного мониторинга в амбулаторных или домашних условиях выглядит перспективным использование малогабаритных портативных устройств, не требующих сложной пробоподготовки биоматериала и предоставляющих результат анализа в течение нескольких минут [7].

В данном обзоре рассмотрены основные патологии, при которых рекомендуется определять уровень кальция крови, возможности и ограничения применяемых в настоящее время лабораторных методов исследования кальциемии, освещены новые технологические подходы к измерению ионизированного кальция крови.

## ОСНОВНЫЕ СОСТОЯНИЯ, ТРЕБУЮЩИЕ КОНТРОЛЯ ПОКАЗАТЕЛЕЙ КАЛЬЦИЕМИИ

Патология околощитовидных желез (ОЩЖ) является одной из особо значимых причин отклонения показателей кальциемии от нормы. Так, наиболее частой причиной гиперкальциемии является гиперпаратиреоз [8]. По данным эпидемиологического исследования в Соединенных Штатах Америки, включавшем 3,5 млн пациентов и длившемся в период с 1995 по 2010 гг., распространенность первичного гиперпаратиреоза (ПГПТ) составила 233 и 85 человек на 100 тыс. населения среди женщин и мужчин соответственно [9]. Масштабных популяционных исследований распространенности ПГПТ в России не проводилось, однако с 2016 г. ведется российский регистр ПГПТ, и, согласно опубликованным данным, на 2022 г. в базе содержались сведения о 6003 пациентах с данным заболеванием [10]. При длительном течении ПГПТ на фоне нарушения фосфорно-кальциевого обмена развиваются полиорганные осложнения со стороны почек, костной, сердечно-сосудистой, мышечной систем, желудочно-кишечного тракта, что приводит к снижению качества жизни пациентов [11]. Для верификации диагноза ПГПТ и назначения терапии важна лабораторная диагностика, включающая определение уровней кальция и ПТГ в крови, а также фосфора и витамина D [12]. Выявление гиперкальциемического варианта бессимптомного ПГПТ чаще всего происходит по результатам рутинного скрининга кальция [11]. Например, в описанном выше американском исследовании путем повторных измерений была обнаружена хроническая гиперкальциемия у более 15 тыс. человек (0,4%), в 87% случаев (13 327 человек) обусловленная ПГПТ, протекавшим бессимптомно [9]. В Российской Федерации, по данным скрининга кальциемии среди жителей Химок и Коломны Московской области (n=674), проводившемся в 2017 г., частота гиперкальциемии составила 0,7% [13].

Согласно Федеральным клиническим рекомендациям по первичному гиперпаратиреозу (2020 г.), пациентам с бессимптомным течением заболевания и отсутствием показаний к хирургическому лечению при выборе консервативной тактики рекомендуется контроль уровня кальция крови 2–4 раза в год, а установление такого диагноза, как нормокальциемический ПГПТ, требует минимум двукратного измерения показателей кальция и ПТГ за период 3–6 месяцев [12].

Противоположной патологией ОЩЖ является гипопаратиреоз, при котором наблюдается недостаточная выработка ПТГ, низкая концентрация кальция крови и высокая — фосфора [14]. Пациентам с гипопаратиреозом требуется динамический мониторинг кальция, фосфора, магния крови, а также креатинина с расчетом скорости клубочковой фильтрации (СКФ) с частотой от 1 раза в 3–6 месяцев при достижении компенсации до нескольких раз в неделю при подборе адекватной терапии. Беременным с гипопаратиреозом рекомендуется регулярно оценивать показатели фосфорно-кальциевого обмена с интервалом в 3–4 недели [15]. В сложных клинических случаях хронического гипопаратиреоза может быть полезным определение суточного профиля кальциемии, так как стандартный подход с измерением уровня кальция в крови в утренние часы не всегда отражает его истинные колебания в течение суток [16]. В литературе описана серия клинических случаев, в которых исследование кальциемии каждые 2 часа в течение суток позволило оценить течение заболевания, верно интерпретировать симптомы и скорректировать лечение [16].

Выраженные нарушения фосфорно-кальциевого обмена наблюдаются при длительном течении ХБП. На фоне снижения экскреции фосфора с мочой и стойкой гиперфосфатемии происходит ингибирование α-1 гидроксилирования 25-гидроксивитамина D, развитие гипокальциемии. Повышается продукция ПТГ, и в результате хронической гиперфункции ОЩЖ возникает их диффузная гиперплазия, что является причиной вторичного гиперпаратиреоза [17]. При длительно персистирующем вторичном гиперпаратиреозе возможно формирование диффузно-узелковой гиперплазии ОЩЖ с развитием третичного гиперпаратиреоза, характеризующимся функциональной автономией ОЩЖ с избыточной секрецией ПТГ и гиперкальциемией [8, 17]. Указанные состояния требуют мониторинга кальций-фосфорного обмена, и, согласно Практическим рекомендациям Инициативы по улучшению глобальных исходов лечения пациентов с ХБП (Kidney Disease: Improving Global Outcomes, KDIGO) по диагностике, профилактике и лечению минеральных и костных нарушений при ХБП (ХБП-МКН), частота измерения уровней кальция и фосфора крови варьирует в пределах 1–6 месяцев, а частота определения уровня ПТГ — 3–12 месяцев, в зависимости от стадии ХБП [18].

При гипокальциемии повышается нервно-мышечная возбудимость и риск судорожного синдрома [19]. При этом пароксизмальные неврологические состояния могут быть проявлением эпилепсии, травмы головного мозга, а также интоксикации, метаболических нарушений, а из электролитных сдвигов наибольшее значение в возникновении острых симптоматических судорог имеет гипокальциемия, гипомагниемия и изменение уровня натрия [20]. Существует значительный риск ошибочного установления диагноза «Эпилепсия» на фоне гипокальциемических состояний [20][21]. Если судорожный синдром, вызванный нарушением гомеостаза других электролитов, чаще возникает вследствие острой патологии (массивная рвота, диарея, передозировка лекарственных средств), то гипокальциемические судороги в большинстве случаев ассоциированы с хронической патологией и повторяются с различной периодичностью [22]. Li et al. (2018 г.) в своей работе приводят данные о гипердиагностике эпилепсии у 179 пациентов, в действительности страдающих гипопаратиреозом, за период с 1994 по 2013 гг. в Китае [23]. В педиатрической практике также актуальна проблема установления этиологии судорог у детей со спазмофилией, генетически обусловленными нарушениями фосфорно-кальциевого обмена, в частности псевдогипопаратиреозом и аутоиммунным полигландулярным синдромом 1-го типа [19][20]. Для адекватной оценки клинической ситуации и своевременного назначения лечения рекомендуется всем детям с судорожным синдромом определять уровень кальция в крови [19].

Отдельного внимания заслуживает гиперкальциемия при злокачественных заболеваниях (ГКЗНО), встречающаяся у 2–30% пациентов с онкологической патологией [24]. Чаще всего ГКЗНО возникает при таких солидных опухолях, как рак молочной железы, легких, почек, а также при множественной миеломе [25]. Наличие ГКЗНО ассоциировано с более тяжелым течением заболевания и неблагоприятным прогнозом [26]. Bhandari et al. (2019 г.) сравнили исходы у 7,5 млн госпитализированных больных с солидными опухолями: в группе с сопутствующей ГКЗНО смертность составила 12,3% в сравнении с 5,5% в группе без ГКЗНО, при этом другие причины гиперкальциемии были исключены [27]. Раннее выявление и коррекция ГКЗНО способствовали бы улучшению клинического прогноза у онкологических пациентов [24][26].

Эффекты патологических изменений фосфорно-кальциевого обмена играют значительную роль в метаболизме костной ткани [4]. При этом дефицит кальция — один из основных факторов риска развития остеопороза [28]. На первичный остеопороз приходится 95% всех случаев остеопороза у женщин в постменопаузе и 80% случаев у мужчин старше 50 лет [29]. Согласно Федеральным клиническим рекомендациям по диагностике, лечению и профилактике остеопороза (2021 г.), в обязательный набор лабораторных исследований крови при впервые установленном остеопорозе включены, помимо стандартных общеклинических показателей, общий магний, неорганический фосфор, общий и ионизированный кальций [29]. Результаты анализов позволяют в том числе и уточнить противопоказания к определенным лекарственным средствам. В частности, при гипокальциемии противопоказано назначение бисфосфонатов и деносумаба, при гиперкальциемии — терипаратида [29][30]. Также важным клиническим аспектом является наличие коморбидной патологии у многих пациентов с нарушениями фосфорно-кальциевого обмена, что может искажать истинные значения кальциемии при определении уровня общего кальция крови в сравнении с измерением ионизированного [31].

## ИОНИЗИРОВАННЫЙ И АЛЬБУМИН-СКОРРЕКТИРОВАННЫЙ КАЛЬЦИЙ КРОВИ

Из общего количества циркулирующего кальция в крови только около 50% является биологически активной ионной формой, остальная часть связана с анионами (10–15%), глобулинами и альбумином (35–40%) [32]. В практической деятельности необходимо оценивать концентрацию свободной фракции в плазме, и для этого используются две альтернативы:

1) общий кальций,

2) ионизированный кальций.

При измерении общего кальция плазмы необходимо параллельно определять уровень альбумина и затем рассчитывать альбумин-скорректированный кальций [15][32]. Корректирование общего кальция по альбумину чаще всего осуществляют по формуле Пейна:

Ca коррект = Ca плазмы + 0,02 × [ 40 – альбумин]

Примечание: Ca коррект — скорректированный кальций (ммоль/л), Ca плазмы — измеренный уровень кальция плазмы (ммоль/л), альбумин — измеренный уровень альбумина плазмы (г/л).

В литературе упоминаются и другие уравнения для расчета, а также описывается практика отдельных лабораторий использовать собственные локальные корректировочные формулы [31–33]. Однако расчетный метод определения концентрации свободного кальция крови по уровню общего с поправкой на альбумин не является эталонным и может привести к некорректной оценке кальциемии [31][33]. Наличие у пациента таких нарушений, как сниженная функция почек (СКФ<30 мл/мин/1,73 м²), уменьшение концентрации сывороточного альбумина (<35 г/л), смещение показателя pH крови приводит к существенной погрешности при использовании формул [31]. При снижении СКФ и/или уровня альбумина скорректированные значения кальциемии получаются завышенными более чем в 50% случаев. На фоне алкалоза рассчитанные данные концентрации кальция также выше истинных и, наоборот, ниже — при ацидозе [31]. В то же время у пациентов с нарушениями фосфорно-кальциевого обмена поражение почек со снижением СКФ встречается достаточно часто, в частности у когорты с патологией ОЩЖ [14][34].

Другим фактором, способствующим искажению вычисляемого уровня кальция крови, является суммация погрешностей при лабораторном анализе концентраций общего кальция и альбумина [35]. Jassam et al. (2020 г.) провели оценку измерений альбумина и общего кальция, полученных в более 100 лабораториях Великобритании на протяжении 6 месяцев, и выявили вариабельность погрешности при определении альбумина от +5,1 до -4,3% и от +2 до -6,7% в зависимости от использования колориметрического метода с бромкрезоловым пурпурным или бромкрезоловым зеленым соответственно [36]. Погрешность измерений общего кальция варьировала от +1,5 до -1%, а на отдельном оборудовании от +3 до -6%. При этом допустимой погрешностью при определении концентрации альбумина считалось значение 1,4% и общего кальция — 1%. При расчете скорректированного кальция по данным измерениям погрешность составила 11% (от +5 до -6%), что эквивалентно размаху 0,24–0,29 ммоль/л и является существенным с учетом узкого референсного интервала 2,2–2,6 ммоль/л [36][37].

Определение вариабельности значений общего кальция крови проводилось и в рамках крупных популяционных исследований. Schini et al. (2021 г.) проанализировали результаты измерений общего кальция с поправкой на связывание с альбумином у 178,4 тыс. человек из когорты биобанка Великобритании (UK Biobank), соответствовавших критериям включения, в том числе СКФ>60 мл/мин/1,73 м² и уровень витамина D>50 нмоль/л [38]. На начальном этапе дополнительно были исключены еще 2961 человек (1,66%) с резко отличающимися показателями кальциемии от основной массы наблюдений (разброс значений от 1,12 ммоль/л до 3,51 ммоль/л). По результатам работы в исследуемой популяции был определен референсный интервал для альбумин-скорректированного кальция 2,19–2,56 ммоль/л.

В свою очередь, определение ионизированного кальция крови также имеет ограничения, к которым относятся высокая стоимость анализаторов, часто возникающая неисправность ионоселективных электродов, зависимость результатов измерений от pH образцов и использованного антикоагулянта (гепарин — антикоагулянт выбора, цитрат натрия и этилендиаминтетрауксусная кислота (ЭДТА) в большей степени связывают ионы кальция) [37][38]. В связи с тем, что методики определения как общего, так и ионизированного кальция до сих пор не являются исчерпывающими, мнения экспертов об их применении в практике разнятся [31][39–41]. При этом имеются преимущества измерения ионизированной формы кальция в отдельных клинических ситуациях, например, при прогнозировании развития гипокальциемии в послеоперационном периоде у перенесших тиреоидэктомию пациентов [42]. Также Tee et al. (2013 г.) установили, что концентрация ионизированного кальция коррелирует с уровнем ПТГ и размером аденом ОЩЖ, и является более чувствительным маркером тяжести течения ПГПТ по сравнению к значению общего кальция [43]. Тем не менее, согласно национальным и зарубежным рекомендациям, в клинической практике в качестве основного метода предлагается определение общего кальция с коррекцией на альбумин при уровне последнего менее 40 г/л и более 45 г/л [12][15][24][29][41].

## ЛАБОРАТОРНЫЕ МЕТОДЫ ОПРЕДЕЛЕНИЯ КОНЦЕНТРАЦИИ КАЛЬЦИЯ В КРОВИ

В настоящее время для определения концентрации общего кальция в крови в клинических лабораториях чаще всего используется фотометрический метод анализа [37]. В его основе лежит цветная реакция, происходящая при селективном связывании кальция с металлохромами и измеряемая на спектрофотометре. Наиболее широко применяются такие индикаторы на кальций, как о-крезолфталеин и арсеназо III. Данные методики чувствительны к отклонениям pH и температуре образцов, что требует тщательного соблюдения требований преаналитического этапа исследований. Более точным методом определения общего кальция крови является атомная абсорбционная спектрометрия (ААС), используемая лишь в отдельных лабораториях в качестве референсного метода [44]. Принцип ААС заключается в селективной абсорбции электромагнитного излучения определенной длины волны свободными атомами исследуемого вещества, находящихся в газообразном состоянии. Исследования Yan et al. (2016 г.) и Zhao et al. (2018 г.) выявили, что самым достоверным методом определения кальция сыворотки крови является масс-спектрометрия с индуктивно связанной плазмой [45][46]. Специальным образом подготовленный раствор исследуемого образца подается в распылитель и в виде аэрозоля передается в аргоновую плазму, где под воздействием высокой температуры диссоциирует на атомы и ионизируется, затем образовавшиеся ионы направляются на детектор. Однако проведение данного вида анализа требует значительных финансовых затрат, тщательной пробоподготовки и дополнительного обучения персонала, что затрудняет его использование в практическом здравоохранении.

Прямые методы определения ионизированного кальция крови основаны на использовании различных модификаций электродов [47]. Классическим и наиболее простым вариантом являются ионоселективные электроды, применяемые в стационарных лабораторных анализаторах, работающих с пробоподготовленной плазмой. В последние годы наблюдается тенденция к упрощению и ускорению процедуры анализа [48]. Так, был разработан ряд портативных устройств Point-of-Care (POC) [49]. Многие модели приборов POC среди прочих показателей измеряют уровень ионизированного кальция крови. Наиболее известными вариантами подобных аппаратов являются Abbott i-STAT Chem8+® и Nova Stat Profile Prime Plus® [50]. Устройства в основном используются в отделениях интенсивной терапии и реанимации, время проведения анализа составляет 1,5–2 минуты, для процедуры требуется 95–135 мкл цельной венозной крови. Принцип работы основан на детекции исследуемых ионов или веществ микроэлектродами, объединенными в биосенсор в виде микрокартриджей.

Также перспективным для динамического наблюдения за пациентами с нарушениями фосфорно-кальциевого обмена в качестве домашнего мониторинга могло бы стать создание моделей POC, изолированно определяющих уровень кальция [6][7]. В настоящее время подобные устройства исследовались в ветеринарии с целью решения проблемы своевременной диагностики послеродовой гипокальциемии у коров, однако точность анализа в этих приборах остается недостаточной [51–53]. В том числе имеются технические сложности, например, ионоселективные электроды, рассчитанные на многоразовое использование, быстро выходят из строя при контакте с кровью [37]. Одним из решений является защита электродов полимерной мембраной, и ее наиболее оптимальный состав находится в разработке [54]. Более перспективный вариант — использование одноразовых биосенсоров по типу тест-полосок или микрокартриджей по аналогии с устройствами POC, применяющихся в отделениях интенсивной терапии [37].

Развивающимся направлением современной диагностики являются модификации биосенсоров, предназначенные для ношения на поверхности тела (“wearable biosensors”) [47]. Такие датчики позволяют проводить неинвазивный мониторинг интересующего параметра в крови и иных биологических жидкостях. Многие модели устройств интегрированы с программным обеспечением смартфона, с которым связываются при помощи Bluetooth (рис. 1). В настоящее время биосенсоры для мониторинга уровня ионизированного кальция в клинической практике не применяются, но существуют в рамках экспериментальных работ. Так, Nyein et al. (2016 г.) создали биосенсор, определяющий в режиме реального времени концентрацию ионизированного кальция и значение рН в поте, планируются дальнейшие разработки для других биологических жидкостей [55]. Подобный неинвазивный метод оценки кальциемии был бы особенно актуален в педиатрической практике. Биосенсор на основе микроигл, сконструированный Molinero-Fernández et al. (2023 г.), позволяет мониторировать уровень ионизированного кальция непосредственно в крови, параллельно с определением pH и ионов K, Na, Li, Cl [56]. Но исследование данного прибора осуществлялось только в лабораторных условиях на животных.

**Figure:**
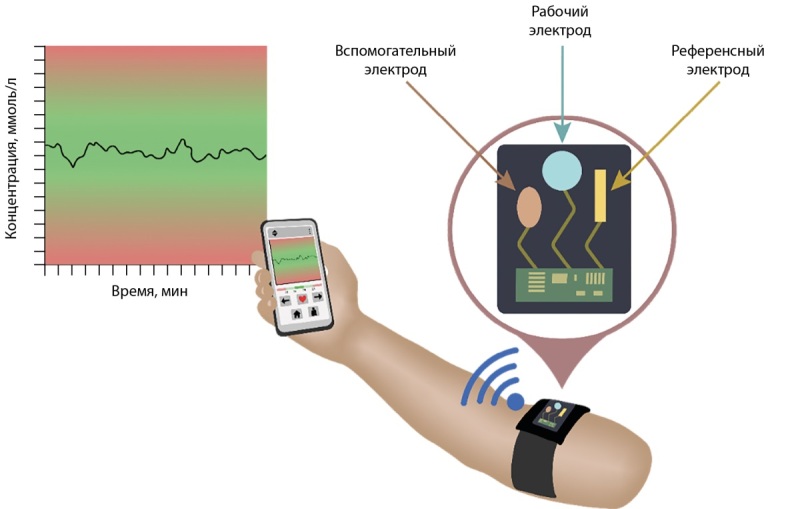
Рисунок 1. Общий принцип работы нательного биосенсора (“wearable biosensor”).

Методы определения общего и ионизированного кальция наглядно представлены в таблице 1.

**Table:** Таблица 1. Методы для определения общего и ионизированного кальция в крови

Общий кальций	Ионизированный кальций
Фотометрия • индикатор о-крезолфталеин • индикатор арсеназо III	Лабораторные анализаторы с ионоселективными электродами
Атомная абсорбционная спектрометрия	Биосенсоры • устройства Point-of-Care • нательные биосенсоры
Масс-спектрометрия с индуктивно связанной плазмой
Референсный интервал: 2,2–2,6 ммоль/л [35]	Референсный интервал: 1,12–1,32 ммоль/л [58]

## ЗАКЛЮЧЕНИЕ

Способы определения кальциемии в последнее время были усовершенствованы в сторону повышения точности лабораторного анализа, также появляются новые портативные приборы, предоставляющие результаты в течение нескольких минут. Данный подход в диагностике позволяет врачам быстро выбрать верную тактику лечения больных в сложных клинических ситуациях [7].

Мониторинг ионизированного кальция в крови с помощью устройств POC или нательных биосенсоров может быть полезным для пациентов с критическими нарушениями фосфорно-кальциевого обмена, угрожающими развитием острых состояний. Также частая оценка позволит наблюдать более точную картину гомеостаза кальция и, возможно, изменит подходы к лечению и дозированию лекарственных средств. Например, подобный контроль показателей выглядит перспективным для больных с гипопаратиреозом, особенно на период подбора терапии до достижения индивидуальных целевых показателей кальция крови [6]. Использование портативных неинвазивных приборов может иметь преимущества и в ранней диагностике нарушения функции ОЩЖ в послеоперационном периоде у пациентов с хирургическими вмешательствами на шее [57]. Скрининг на нарушение кальциевого обмена требуется и при спастическом синдроме, ХБП, нефролитиазе, необъяснимыми другими причинами полидипсии с полиурией, удлинении или увеличении интервала QT электрокардиограммы, онкологии, патологии костной ткани [2]. Тем не менее необходимы дальнейшие исследования медицинских изделий для адаптации новых технологий к реалиям клинической практики.

## ДОПОЛНИТЕЛЬНАЯ ИНФОРМАЦИЯ

Источники финансирования. Работа выполнена по инициативе авторов без привлечения финансирования.

Конфликт интересов. Авторы декларируют отсутствие явных и потенциальных конфликтов интересов, связанных с содержанием настоящей статьи.

Участие авторов. Все авторы одобрили финальную версию статьи перед публикацией, выразили согласие нести ответственность за все аспекты работы, подразумевающую надлежащее изучение и решение вопросов, связанных с точностью или добросовестностью любой части работы.
